# Blockade or deletion of transient receptor potential vanilloid 4 (TRPV4) is not protective in a murine model of sepsis

**DOI:** 10.12688/f1000research.6298.1

**Published:** 2015-04-20

**Authors:** Claire A. Sand, Anna Starr, Manasi Nandi, Andrew D. Grant

**Affiliations:** 1 William Harvey Research Institute, Barts and The London School of Medicine, Queen Mary University of London, London, EC1M 6BQ, UK; 2 Institute of Pharmaceutical Science, King's College London, London, SE1 9NH, UK; 3 Wolfson Centre for Age-Related Diseases, King's College London, London, SE1 1UL, UK

**Keywords:** TRPV4, Sepsis, Endotoxaemia, Mouse model, Vascular dysfunction, Blood flow, Haemodynamics

## Abstract

Sepsis is a systemic inflammatory response triggered by microbial infection that can cause cardiovascular collapse, insufficient tissue perfusion and multi-organ failure. The cation channel transient receptor potential vanilloid 4 (TRPV4) is expressed in vascular endothelium and causes vasodilatation, but excessive TRPV4 activation leads to profound hypotension and circulatory collapse - key features of sepsis pathogenesis. We hypothesised that loss of TRPV4 signaling would protect against cardiovascular dysfunction in a mouse model of sepsis (endotoxaemia).

Multi-parameter monitoring of conscious systemic haemodynamics (by radiotelemetry probe), mesenteric microvascular blood flow (laser speckle contrast imaging) and blood biochemistry (iSTAT blood gas analysis) was carried out in wild type (WT) and TRPV4 knockout (KO) mice. Endotoxaemia was induced by a single intravenous injection of lipopolysaccharide (LPS; 12.5 mg/kg) and systemic haemodynamics monitored for 24 h. Blood flow recording was then conducted under terminal anaesthesia after which blood was obtained for haematological/biochemical analysis. No significant differences were observed in baseline haemodynamics or mesenteric blood flow. Naïve TRPV4 KO mice were significantly acidotic relative to WT counterparts. Following induction of sepsis, all mice became significantly hypotensive, though there was no significant difference in the degree of hypotension between TRPV4 WT and KO mice. TRPV4 KO mice exhibited a higher sepsis severity score. While septic WT mice became significantly hypernatraemic relative to the naïve state, this was not observed in septic KO mice. Mesenteric blood flow was inhibited by topical application of the TRPV4 agonist GSK1016790A in naïve WT mice, but enhanced 24 h following LPS injection. Contrary to the initial hypothesis, loss of TRPV4 signaling (either through gene deletion or pharmacological antagonism) did not attenuate sepsis-induced cardiovascular dysfunction: in fact, pathology appeared to be modestly exaggerated in mice lacking TRPV4. Local targeting of TRPV4 signalling may be more beneficial than global inhibition in sepsis treatment.

## Introduction

Sepsis, the systemic inflammatory syndrome that can occur in response to infection, represents an enormous global healthcare burden; it can progress to septic shock, characterised by refractory hypotension and insufficient organ perfusion, with associated mortality rates of up to 80% ( Martin, 2012). However, treatment options for sepsis are currently limited, highlighting the need to refine pre-clinical septic shock models to allow more effective identification of novel drug targets. There is now increasing evidence that several cation-permeable transient receptor potential (TRP) channels, most notably of the vanilloid (TRPV) subfamily, can influence physiological systems compromised in sepsis, and may represent potential therapeutic targets.

TRPV4, originally identified as an osmosensor in the kidney ( Liedtke *et al.*, 2000; Strotmann *et al.*, 2000; Wissenbach *et al.*, 2000), is expressed in numerous tissues, including the heart and vasculature ( Wissenbach *et al.*, 2000). Its expression in both endothelial and smooth muscle cells is well-established, and its activation in vascular tissue is associated with endothelium-dependent vasodilatation, and both direct and indirect smooth muscle hyperpolarisation ( Baylie & Brayden, 2011). In addition to extracellular hypotonicity, it is gated by various physical and chemical stimuli, including shear stress, arachidonic acid metabolites (particularly epoxyeicosatrienoic acids) and endocannabinoids ( Nilius *et al.*, 2004). These chemical factors may be increased under inflammatory conditions ( Kiss *et al.*, 2010; Theken *et al.*, 2011; Varga *et al.*, 1998). Furthermore, previous studies have identified sensitisation of TRPV4 without direct activation by other common inflammatory mediators ( Alessandri-Haber *et al.*, 2006). Recent reports have linked excessive TRPV4 activation to lethal endothelial failure, oedema formation, hypotension and circulatory collapse ( Sonkusare *et al.*, 2012; Willette *et al.*, 2008), suggesting it may play a key role in sepsis pathogenesis. Given that numerous endogenous agonists and sensitisers of TRPV4 are upregulated in sepsis, we hypothesised that blockade of TRPV4 activity using a pharmacological antagonist HC-067047, or genetic deletion of the channel (in global knockout mice) would be protective in a murine model of sepsis, attenuating the cardiovascular collapse associated with the development of septic shock.

## Methods

### Materials

Lipopolysaccharide (LPS) from *Salmonella typhimurium* and the TRPV4 agonist GSK1016790A were purchased from Sigma-Aldrich. The TRPV4 antagonist HC-067047 was purchased from Tocris Bioscience. All other materials were obtained from Sigma-Aldrich, unless otherwise stated.

### Animals

Male mice, either wild type (WT) or littermates lacking functional TRPV4 (KO), were bred in-house from heterozygous breeding pairs originally derived from a colony generated by Liedtke & Friedman ( Liedtke & Friedman, 2003). Mice were maintained on a 12-h light/dark cycle (7am–7pm, and 7pm–7am, respectively) and given access to food (normal chow) and water *ad libitum.* All animal experiments were conducted under a UK Home Office licence (PPL 70/7049), following local ethics committee approval and in accordance with the Home Office Animal (Scientific Procedures) Act, 1986. Experiments were designed and conducted in a blinded manner and in accord with the ARRIVE guidelines ( Kilkenny, 2010). All treatments were randomised. Mice were used between 10 and 14 weeks of age (approx. 25–30g). Individual mice were considered to be independent experimental units. A power calculation on pilot laser perfusion imaging data (α = 0.05; β = 0.2; minimum effect size = 20% alteration in blood flux value) indicated that 6 mice in each experimental group would provide sufficient power.

### Induction of endotoxaemia

LPS-induced endotoxaemia was used as a model for sepsis. Endotoxaemia was induced by intravenous injection of the bacterial endotoxin LPS (12.5 mg/kg). Injections of 100 µl (pre-warmed to 37°C) were administered into the tail vein (using a 29 G needle and insulin syringe) under inhaled isoflurane anaesthesia (2% by air pump). A heating mat set to 37°C was used to dilate the tail vein for improved accessibility. Buprenorphine hydrochloride [15 µg/kg, intramuscular (i.m.)] was administered into the two hind-limbs to provide post-operative analgesia and 0.9% (w/v) saline (20 ml/kg) was administered subcutaneously to provide fluid resuscitation. Mice were placed in a recovery chamber at 27°C for up to 24 h and were monitored frequently. An arbitrary severity score of 1–5 (based on mobility, facial expression, piloerection and aversion to touch) was employed to assess animal welfare. Any mouse reaching a score of 5 was immediately euthanised. All scoring was conducted by an experimenter blind to mouse genotype and treatment.

### Multi-parameter monitoring

**Figure 1.**
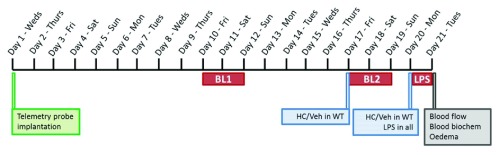
Timeline of experimental procedures. Mice were implanted with telemetry probes on day 1, and were left to recover for 10 days. Basal haemodynamic data were then recorded over a 48-h weekend period (BL1). The following weekend, WT mice were injected with either vehicle (10% DMSO in saline) or TRPV4 antagonist HC-067047 (10 mg/kg, i.p.) and a second 48-h baseline recording (BL2) was taken for all mice. TRPV4 KO mice were left untreated. Injections in WT mice were performed in a randomised and blinded fashion. Following the second weekend baseline recording, WT mice received a second injection of either HC-067047 or vehicle (consistent with initial treatment) and LPS (12.5 mg/kg, i.v.) was administered to all mice. Haemodynamic data were recorded over 24-h of endotoxaemia progression. Mice were then terminally anaesthetised for blood flow recording and *ex vivo* analysis. A separate cohort of naïve mice were included in blood flow and *ex vivo* studies.

A multi-parameter approach, encompassing haemodynamic, microcirculatory and haematological assessment, was used to determine the impact of TRPV4 blockade on endotoxaemia pathophysiology, as described previously ( Sand *et al.*, 2015). A timeline summarising the sequence of experimental procedures is provided in Figure 1.

### Haemodynamic recording

PA-C10 telemetry probes (Data Sciences International; DSI, USA) were implanted into 10–12-week-old mice weighing 25–30g, under inhaled isoflurane anaesthesia using aseptic technique. Mice were placed in the supine position on a homeothermically-controlled heating mat (Harvard Instruments), and the thorax was shaved and disinfected with chlorhexidine. Buprenorphine hydrochloride (15 µg/kg, i.m.) was administered into the two hind-limbs to provide post-operative analgesia, and eyes were protected with Viscotears. A small thoracic incision was made and the carotid artery was isolated by gentle blunt dissection. Telemeter catheters were advanced into the left carotid artery and secured with three silk sutures. The body of the telemetry probe was placed in a subcutaneous pocket in the flank of the animal, and the incision was closed using a 5.0 Vicryl suture. Mice were then placed in a recovery cabinet at 27°C for 4 h, before being moved to a holding room, where they were allowed to recover for at least 7 days before the start of haemodynamic monitoring. The viability of each haemodynamic trace was checked, and mice with significant dampening or loss of signal were excluded from analysis (two in this study). All implantation, monitoring and data analysis were conducted by an experimenter blind to mouse genotype and treatment, and assignment of telemetry probes was randomised.

Following recovery, baseline haemodynamic parameters were recorded over a 48-h weekend period. Food and water intake was measured continuously, and mice were closely monitored for adverse signs. After baseline recordings, the TRPV4 antagonist HC-067047 [10 mg/kg in 500 µl saline, intraperitoneal (i.p.)] or vehicle (10% dimethyl sulfoxide, DMSO, in 500 µl saline, i.p.) was administered in a blinded and randomised fashion to WT mice only. KO mice were left untreated. Baseline haemodynamic parameters were then recorded over a second 48-h weekend period. On the following Monday, TRPV4 WT mice were re-injected with either HC-067047 or vehicle (the same as the first treatment), and endotoxaemia was induced by intravenous administration of LPS. Haemodynamic parameters were recorded for a further 24 h, and mice were then terminally anaesthetised for blood flow assessment.

PA-C10 telemetry probes allow continuous and remote monitoring of blood pressure, heart rate and locomotor activity in freely moving conscious animals. Data were acquired continuously at 500 Hz using standard acquisition software (DSI, USA). Baseline recording was carried out over weekends in order to minimise disruption.

### *In situ* monitoring of mesenteric blood flow

In light of evidence suggesting that microvascular circulation is more prognostic in sepsis than global haemodynamics ( De Backer *et al.*, 2002; Sakr *et al.*, 2004), we analysed mesenteric blood flow in TRPV4 WT and KO mice, both at baseline and during endotoxaemia. The mesenteric bed was chosen for assessment of microcirculatory function for its accessibility and its significant contribution to peripheral vascular resistance.

Mesenteric blood flow was measured in naïve and endotoxaemic mice under inhaled isoflurane anaesthesia (2% delivered by air pump), as described previously ( Sand *et al.*, 2015). Core temperature was recorded and controlled by a rectal probe coupled to a homeothermic heating mat (Harvard Instruments); mice were maintained at the temperature at which they initially presented. The abdominal region was shaved, and a small midline incision was made. A portion of the small intestine was gently exteriorised onto a Parafilm-coated heating mat, and was pinned out through the gut wall to expose the mesenteric vasculature. The exposed vascular bed was kept moist with saline (0.5 ml, aerosolised) pre-warmed to 37°C. An additional heating mat was placed over the anaesthetised animal.

Mesenteric blood flow was recorded using a moorFLPI full-field laser perfusion imaging system and review software (Moor Instruments, Devon, UK) either in naïve animals, or at 6 or 24 h after the induction of endotoxaemia. The following acquisition modes and settings were used: high resolution capture (25 frames, 1 s/frame); exposure 20 ms; automatic gain; flux palette set at 0–5000; background threshold 60 flux units. Vessels within the field of view were designated as 1 ^st^, 2 ^nd^ or 3 ^rd^ order branches of the mesenteric tree, and regions of interest, in which flux over time was measured, were defined in each visible vessel. All assessment and analysis was performed by an experimenter blind to mouse genotype and treatment. Baseline recordings were taken over 5 min, after which the mesenteric bed was gently sprayed (two pump compressions from a distance of approximately 10 cm) with selective TRPV4 agonist GSK1016790A (1 µM) dissolved in saline containing 1% DMSO, and pre-warmed to 37°C. Responses to GSK1016790A were measured over a further 5 min. Data were analysed in moorFLPI Review software (V 4.0; Moor Instruments, UK) and GraphPad Prism (V 5.0; GraphPad Software Inc, USA), and are expressed as mean area under the curve (AUC) over time ± SEM.

### Analysis of blood biochemistry

Following blood flow recording, a venous blood sample was drawn from the inferior vena cava, and animals were killed by cervical dislocation. Blood biochemistry was assessed immediately from 100 µl venous blood using a hand-held iSTAT ^®^ point-of-care analyser (Abbott Laboratories, IL, USA), with CG8+ cartridges (Abbott Laboratories).

### Assessment of organ oedema

Oedema formation was measured by comparing wet and dry weights of lungs, hearts, upper liver lobes, right kidneys, 1 cm sections of small intestine and whole spleens. Tissues were excised immediately after blood flow recording and wet weights were recorded. Samples were then placed in an oven at 50°C until a constant dry weight was reached (approximately 3 days). The ratio of wet weight: dry weight was used as an indication of oedema formation.

### Statistical analysis

Statistical analysis was conducted in GraphPad Prism. Mean data for ‘n’ number of animals were compared by 1-way ANOVA with Bonferroni post-hoc test, unless stated otherwise. The threshold for statistical significance was p < 0.05.

## Results

**Figure 2.**
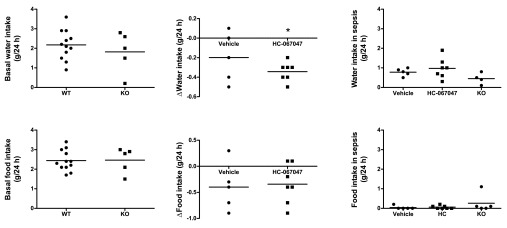
Water and food intake in TRPV4 WT and KO mice before and after induction of endotoxaemia. ( **a**) Mean basal water intake in TRPV4 WT and KO mice over approximately 1 week (not including 7-day recovery period from telemetry surgery). ( **b**) Change in water intake over 24-h period following i.p. administration of either vehicle (10% DMSO) or HC-067047. ( **c**) Water intake over 24-h period following administration of LPS (12.5 mg/kg i.v.). ( **d**) Mean basal food intake in TRPV4 WT and KO mice over approximately 1 week (not including 7-day recovery period from telemetry surgery). ( **e**) Change in food intake over 24-h period following i.p. administration of either vehicle (10% DMSO) or HC-067047. ( **f**) Food intake over 24-h period following administration of LPS (12.5 mg/kg i.v.). Data for individual animals are represented as individual symbols, with group mean denoted by horizontal line. *p<0.05, relative to vehicle-treated controls, 1-way ANOVA + Bonferroni post-hoc test, n = 5–12.

TRPV4 WT and KO mice were singly-housed from the time of radiotelemeter implantation throughout the duration of the study. Food and water intake were monitored throughout. Recovery from telemetry surgery (measured as recovery in body weight, food and water intake and physical examination) was equivalent across all animals. All mice returned to their pre-surgery weight and food water intake within 7 days of implantation. Following recovery, TRPV4 KO mice were found to consume less water than WT counterparts, though this difference was not statistically significant ( Figure 2a). Systemic administration of either HC-067047 or its vehicle to WT mice reduced water intake, though the magnitude of change was significantly greater in HC067047-treated animals ( Figure 2b). Water intake was reduced in all animals over the 24 h following the induction of endotoxaemia ( Figure 2c). No difference in food intake was observed between different genotypes basally ( Figure 2d). Systemic administration of either vehicle or HC-067047 reduced food intake, though to a similar degree across treatment groups ( Figure 2e). Induction of sepsis with LPS [12.5 mg/kg, intravenous (i.v.)] markedly reduced food intake to a similar degree in all groups ( Figure 2f).

Raw Data for Figure 2 Water and food intake in TRPV4 WT and KO mice before and after induction of endotoxaemia. Basal water intake was measured in TRPV4 WT and KO mice over approximately 1 week (not including 7-day recovery period from telemetry surgery). Change in water intake was measured over 24-h period following i.p. administration of either vehicle (10% DMSO) or HC-067047, as well as during the 24-h period following administration of LPS (12.5 mg/kg i.v.). Basal food intake in TRPV4 WT and KO mice was measured over approximately 1 week (not including 7-day recovery period from telemetry surgery). Change in food intake was measured over 24-h period following i.p. administration of either vehicle (10% DMSO) or HC-067047, as well as during the 24-h period following administration of LPS (12.5 mg/kg i.v.) ( Sand *et al.*, 2015a).

**Figure 3.**
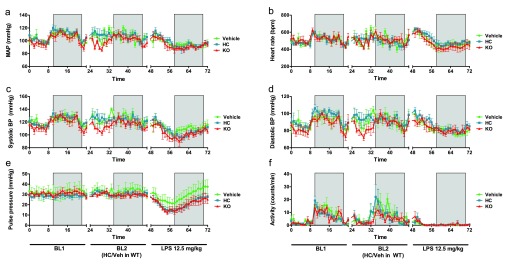
Haemodynamic monitoring in TRPV4 WT and KO mice under basal and endotoxaemic conditions. ( **a**) Mean arterial pressure, ( **b**) heart rate, ( **c**) systolic pressure, ( **d**) diastolic pressure, ( **e**) pulse pressure and ( **f**) locomotor activity at baseline (BL1), following systemic treatment (WT mice only) with HC-067047 (HC) or vehicle (10% DMSO) (BL2) and after induction of endotoxaemia (at time 48 h: LPS, 12.5 mg/kg, i.v.). Boxed regions denote periods of darkness. Data were acquired by radiotelemetry in conscious, ambulatory male C57BL/6 mice, and are presented as mean ± SEM, n = 4–6.

Under basal conditions, all animals exhibited diurnal variation in blood pressure, heart rate and ambulatory activity ( Figure 3), within the expected physiological range for mice ( Curtis *et al.*, 2007). No significant difference in basal haemodynamic parameters was observed between genotypes, although TRPV4 KO mice exhibited a trend towards lower blood pressure (both daytime and night-time) and lower daytime ambulatory activity than WT counterparts [average mean arterial pressure (MAP) over 24 h: KO = 93.71 ± 2.83 mmHg vs. WT = 99.89 ± 1.87 mmHg, ns; mean daytime activity over 12 h: KO = 3.25 ± 0.43 counts/min vs. WT = 4.31 ± 0.33 counts/min, ns (n = 5–10)] ( Figure 3a,c,d,f).

Systemic administration of the TRPV4 antagonist HC-067047 caused an increase in mean daytime blood pressure of approximately 5 mmHg, though this was reversed after 12 h, leading to small but consistent decrease in night-time pressure ( Table 1). Systemic administration of vehicle (10% DMSO in saline) also caused an increase in blood pressure, though this was more transient ( Table 1 & Figure 3). Neither administration of HC-067047 nor of vehicle significantly altered any other haemodynamic parameters under basal conditions, though heart rate was transiently increased relative to time-matched pre-treatment values in both groups, and subsequently declined during night-time ( Table 1). In both cases (daytime tachycardia and night-time bradycardia) the magnitude of the change was greater in HC-067047-treated mice than in vehicle-treated controls, though this was not statistically significant. Mice treated with HC-067047 did exhibit significantly greater daytime ambulatory activity, relative to time-matched pre-treatment values ( Table 1); this change was not observed in vehicle-treated animals, and was no longer evident during night-time hours.

**Table 1.** Statistical comparison of haemodynamic parameters in TRPV4 WT and KO mice under basal and endotoxaemic conditions. Data are presented as mean difference (95% CI), and are divided into daytime and night-time phases. BL1 represents mean baseline values in untreated mice over 48-h weekend recording. BL2 represents mean baseline values following i.p. treatment (WT mice only) with vehicle (10% DMSO) or HC-067047; BL2 in KO mice represents a separate untreated 48-h baseline recording. LPS represents mean values following systemic treatment with LPS (12.5 mg/kg, i.v.) over 24-h recording period. *p<0.05, **p<0.01, ***p<0.001, 1-way ANOVA + Bonferroni post-hoc test.

**Mean arterial pressure, mean diff (95% CI), mm Hg**						
	*Day*			*Night*		
Comparison	**WT + Vehicle** (n = 4)	**WT + HC-067047** (n = 6)	**TRPV4 KO** (n = 5)	**WT + Vehicle** (n = 4)	**WT + HC-067047** (n = 6)	**TRPV4 KO** (n = 5)
BL1 – BL2	+4.835 (-10.99 to +20.66)	+5.297 (-7.62 to +18.21)	-0.406 (-14.56 to +13.74)	-1.532 (-13.82 to +10.75)	-4.956 (-14.99 to +5.08)	-2.327 (-13.32 to +8.66)
BL1 – LPS	-2.838 (-18.66 to +12.98)	-2.124 (-15.04 to +10.79)	-4.326 (-18.48 to +9.825)	-14.480** (-29.77 to +2.19)	-19.020*** (-29.05 to -8.99)	-15.410*** (-26.4 to -4.418)
BL2 – LPS	-7.674 (-23.49 to +8.147)	-7.421 (-20.34 to +5.50)	-3.920 (-18.07 to +10.23)	-12.95* (-25.23 to +0.66)	-14.060 (-24.09 to -4.03)	-13.080** (-24.07 to -2.09)
**Systolic pressure, mean diff (95% CI), mm Hg**						
	*Day*			*Night*		
Comparison	**WT + Vehicle** (n = 4)	**WT + HC-067047** (n = 6)	**TRPV4 KO** (n = 5)	**WT + Vehicle** (n = 4)	**WT + HC-067047** (n = 6)	**TRPV4 KO** (n = 5)
BL1 – BL2	+4.523 (-14.82 to +23.87)	+5.443 (-10.35 to +21.24)	-1.898 (-19.20 to +15.41)	-0.935 (-19.33 to +17.46)	-3.393 (-18.41 to +11.62)	-2.923 (-19.37 to +13.53)
BL1 – LPS	-7.558 (-26.90 to +11.79)	-6.681 (-22.48 to +9.12)	-9.699 (-27.00 to +7.61)	-14.64 (-33.03 to +3.75)	-22.590*** (-37.61 to -7.58)	-19.870** (-36.32 to -3.42)
BL2 – LPS	-12.08 (-31.43 to +7.27)	-12.12 (-27.92 to +3.67)	-7.801 (-25.10 to +9.50)	-13.70 (-32.10 to +4.69)	-19.20** (-34.22 to -4.19)	-16.94* (-33.39 to -0.49)
**Diastolic pressure, mean diff (95% CI), mm Hg**						
	*Day*			*Night*		
Comparison	**WT + Vehicle** (n = 4)	**WT + HC-067047** (n = 6)	**TRPV4 KO** (n = 5)	**WT + Vehicle** (n = 4)	**WT + HC-067047** (n = 6)	**TRPV4 KO** (n = 5)
BL1 – BL2	+4.175 (-12.49 to +20.84)	+3.614 (-10.00 to +17.22)	-0.3836 (-15.29 to +14.53)	-1.603 (-13.95 to +10.75)	-6.103 (-16.19 to +3.98)	-0.263 (-11.31 to +10.78)
BL1 – LPS	-0.366 (-17.04 to +16.30)	+1.581 (-12.03 to +15.19)	+2.953 (-11.96 to +17.86)	-13.410* (-25.76 to -1.06)	-15.940*** (-26.03 to -5.86)	-9.740 (-20.79 to +1.307)
BL2 – LPS	-4.541 (-21.21 to +12.13)	-2.033 (-15.64 to +11.58)	+3.337 (-11.57 to +18.25)	-11.810 (-24.16 to +0.54)	-9.841 (-19.93 to +0.24)	-9.476 (-20.52 to +1.57)
**Heart rate, mean diff (95% CI), bpm**						
	*Day*			*Night*		
Comparison	**WT + Vehicle** (n = 4)	**WT + HC-067047** (n = 6)	**TRPV4 KO** (n = 5)	**WT + Vehicle** (n = 4)	**WT + HC-067047** (n = 6)	**TRPV4 KO** (n = 5)
BL1 – BL2	+18.45 (-80.45 to +117.40)	+22.51 (-58.25 to +103.30)	-0.96 (-89.43 to +87.50)	-19.20 (-126.00 to +87.55)	-34.53 (-121.70 to +52.63)	-10.50 (-106 to +84.98)
BL1 – LPS	+23.19 (-75.75 to +122.10)	+56.30 (-24.46 to +137.10)	-2.04 (-90.50 to +86.42)	-57.22 (-164.00 to +49.54)	-42.61 (-129.80 to +44.55)	-86.79 (-182.30 to +8.69)
BL2 – LPS	+4.74 (-94.17 to 103.60)	+33.79 (-46.97 to +114.50)	-1.08 (-89.54 to +87.39)	-38.01 (-144.80 to +68.74)	-8.076 (-95.24 to +79.09)	-76.29 (-171.80 to +19.20)
**Pulse pressure, mean diff (95% CI), mm Hg**						
	*Day*			*Night*		
Comparison	**WT + Vehicle** (n = 4)	**WT + HC-067047** (n = 6)	**TRPV4 KO** (n = 5)	**WT + Vehicle** (n = 4)	**WT + HC-067047** (n = 6)	**TRPV4 KO** (n = 5)
BL1 – BL2	+1.049 (-13.99 to +16.08)	+1.941 (-10.33 to +14.22)	-0.475 (-13.92 to +12.97)	+1.003 (-17.85 to +19.86)	+1.149 (-14.24 to +16.54)	-2.314 (-19.18 to +14.55)
BL1 – LPS	-6.405 (-21.44 to +8.63)	-9.437 (-21.71 to +2.84)	-11.62 (-25.06 to +1.83)	-0.939 (-19.79 to +17.91)	-6.659 (-22.05 to +8.74)	-9.965 (-26.83 to +6.90)
BL2 – LPS	-7.454 (-22.49 to +7.58)	-11.38 (-23.65 to +0.90)	-11.14 (-24.59 to +2.31)	-1.943 (-20.80 to +16.91)	-7.808 (-23.20 to +7.59)	-7.651 (-24.51 to +9.21)
**Activity, mean diff (95% CI), counts/min**						
	*Day*			*Night*		
Comparison	**WT + Vehicle** (n = 4)	**WT + HC-067047** (n = 6)	**TRPV4 KO** (n = 5)	**WT + Vehicle** (n = 4)	**WT + HC-067047** (n = 6)	**TRPV4 KO** (n = 5)
BL1 – BL2	+1.548 (-1.97 to +5.07)	+3.260* (+0.39 to +6.14)	+0.880 (-2.27 to +4.03)	-0.114 (-4.20 to -397)	-0.360 (-3.69 to +2.97)	-2.169 (-3.30 to +3.69)
BL1 – LPS	-3.764 (-7.29 to +0.24)	-2.762 (-5.64 to +0.11)	-1.568 (-4.72 to +1.58)	-8.932*** (-13.02 to -4.85)	-5.788*** (-9.12 to -2.45)	-7.105*** (-10.76 to -3.45)
BL2 – LPS	-5.311*** (-8.83 to +1.79)	-6.023*** (-8.90 to -3.15)	-2.448 (-5.60 to +0.70)	-8.818*** (-12.90 to -4.74)	-5.428*** (-8.76 to -2.09)	-4.936** (-8.59 to -1.28)

Since TRPV4 KO mice were not treated with pharmacological stimuli, baseline parameters were simply re-recorded in the same mice. Values were relatively consistent across separate baseline recording periods ( Table 1), confirming the robustness of the monitoring system.

Systemic administration of LPS caused a significant decline in blood pressure in all animals ( Figure 3a,c,d & Table 1), that began to stabilise after approximately 12 h. No significant difference between genotypes was observed, though both TRPV4 KOs and antagonist-treated mice exhibited a trend towards more severe hypotension, particularly in systolic pressure ( Figure 3a,c and Table 1). Pulse pressure was compromised in TRPV4 KO and HC-067047-treated mice, relative to vehicle-treated controls ( Figure 3e and Table 1).

An immediate increase in heart rate was observed in both HC-067047- and vehicle-treated WT animals following the induction of endotoxaemia, and this tachycardia was sustained during daylight hours. TRPV4 KO mice exhibited a transient increase in heart rate immediately after LPS administration, but became bradycardic thereafter, indicating failure of the baroreceptor reflex ( Figure 3b and Table 1). All mice exhibited marked bradycardia during night-time hours, relative to time-matched pre-LPS values, though the magnitude of the decrease was greatest in TRPV4 KO mice ( Table 1). Ambulatory activity was almost entirely abolished in all mice following the induction of endotoxaemia ( Figure 3f).

Raw Data for Figure 3 Haemodynamic monitoring in TRPV4 WT and KO mice under basal and endotoxaemic conditions. Values were taken following systemic treatment (WT mice only) with HC-067047 (HC) or vehicle (10% DMSO) (BL2) and after induction of endotoxaemia (at time 48 h: LPS, 12.5 mg/kg, i.v.). Boxed regions denote periods of darkness. Data were acquired by radiotelemetry in conscious, ambulatory male C57BL/6 mice ( Sand *et al.*, 2015b).

All mice telemetered for haemodynamic recording were subjected to mesenteric blood flow analysis. A group of naïve animals were included for comparison, and additional vehicle- and HC-067047-treated WT mice were used to increase statistical power.

**Figure 4.**
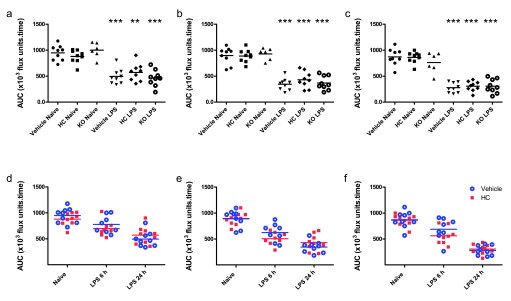
Mesenteric blood flow in healthy and endotoxaemic TRPV4 WT and KO mice. ( **a**– **c**) Mesenteric blood flow in TRPV4 KO mice and WT mice treated i.p. with vehicle (10% DMSO) or HC-067047 (HC) for 24 h, either under naïve or endotoxaemic (LPS 12.5 mg/kg, i.v., 24 h) conditions. Data are expressed as total area under the curve (AUC; ×10 ^3^ flux units.time) over 5-min baseline recording in 1 ^st^, 2 ^nd^ and 3 ^rd^ order vessels, respectively. **p<0.01, ***p<0.001, relative to naïve counterparts, 1-way ANOVA + Bonferroni post-hoc test. ( **d**– **f**) Mesenteric blood flow in WT mice treated i.p. with vehicle (10% DMSO) or HC-067047, either under naïve (24-h treatment) or endotoxaemic conditions (6 h or 24 h; drugs administered simultaneously with LPS) in 1 ^st^, 2 ^nd^ and 3 ^rd^ order vessels, respectively. AUC for each animal is represented as an individual symbol, with horizontal line denoting group mean, n = 6–9.

Mesenteric blood flow was significantly reduced in all groups 24 h after the induction of endotoxaemia ( Figure 4a–c). No significant differences were observed between genotypes or treatment groups, though endotoxaemic mice pre-treated with HC-067047 exhibited a trend towards improved flow. In order to determine whether blockade of TRPV4 may play a role earlier in pathogenesis, mesenteric flow was recorded in a separate cohort of WT mice treated for 6 h with HC-067047 and LPS. At this time-point, blood flow in HC-067047-treated mice was decreased relative to vehicle-treated controls, though these changes were not statistically significant ( Figure 4e–f).

Raw Data for Figure 4 Mesenteric blood flow in healthy and endotoxaemic TRPV4 WT and KO mice. TRPV4 KO mice and WT mice were treated i.p. with vehicle (10% DMSO) or HC-067047 (HC) for 24 h, either under naïve or endotoxaemic (LPS 12.5 mg/kg, i.v., 24 h) conditions ( Sand *et al.*, 2015c).

**Figure 5.**
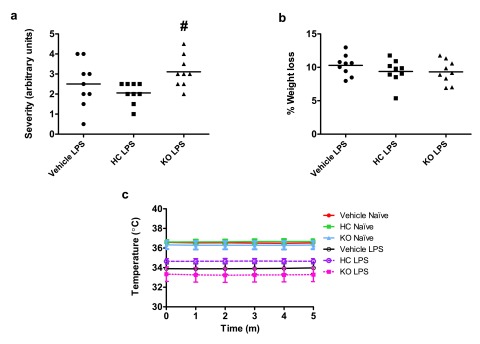
Clinical parameters in TRPV4 WT and KO mice. ( **a**) Arbitrary severity score assigned in a blinded fashion after 24-h treatment with LPS (12.5 mg/kg, i.v.) based on voluntary mobility, gait, aversion to touch, facial expression and piloerection. WT mice were treated i.p. with vehicle (10% DMSO) or HC-067047 (HC) at the time of LPS administration. TRPV4 KO mice did not receive any pharmacological treatment. ( **b**) Percentage weight loss over 24-h endotoxaemic period. Values for each animal are presented as individual symbols, with horizontal line denoting group mean. ^#^p<0.05, relative to HC-treated animals, 1-way ANOVA + Bonferroni post-hoc test (n = 8–9). ( **c**) Core temperature measured by rectal probe throughout blood flow recording period. Data are presented as mean ± SEM (n = 8–9).

Antagonism or gene ablation of TRPV4 had only minor effects on clinical phenotype, though in opposing directions. In line with marginally improved mesenteric perfusion, antagonist-treated mice exhibited a lower sepsis severity score after 24 h treatment with LPS, relative to vehicle-treated controls. On the other hand, the severity score in TRPV4 KO mice was significantly greater than in antagonist-treated counterparts ( Figure 5a). The percentage weight loss over 24 h of endotoxaemia was roughly equivalent between groups, but was lower in antagonist-treated and TRPV4 KO mice, than in vehicle-treated controls ( Figure 5b). Core temperature decreased significantly in all LPS-treated mice, though remained stable in all animals throughout the recording period ( Figure 5c). Although no statistically significant differences in core temperature were observed between groups, there were trends towards attenuated hypothermia in HC-067047-treated mice, and exaggerated hypothermia in TRPV4 KO mice, relative to vehicle-treated counterparts.

Raw Data for Figure 5 Clinical parameters in TRPV4 WT and KO mice. An arbitrary severity score was assigned in a blinded fashion after 24-h treatment with LPS (12.5 mg/kg, i.v.) based on voluntary mobility, gait, aversion to touch, facial expression and piloerection. Percentage weight-loss after endotoxaemic period and core temperature were also measured. WT mice were treated i.p. with vehicle (10% DMSO) or HC-067047 (HC) at the time of LPS administration. TRPV4 KO mice did not receive any pharmacological treatment ( Sand *et al.*, 2015d).

Pharmacological TRPV4 antagonism and gene ablation also appeared to have divergent effects on blood biochemistry ( Table 2). Although the concentration of urea was elevated in all groups 24 h post-LPS, the increase in TRPV4 KO mice was greatest, whereas that in antagonist-treated mice did not reach statistical significance. Blood pH was also lowest in LPS-treated TRPV4 KO mice, whereas acidosis was attenuated in antagonist-treated mice, relative to vehicle-treated controls. PCO _2_ was increased by LPS treatment, and bicarbonate decreased, indicative of respiratory and metabolic acidosis, respectively, though no significant differences were observed across treatment groups. All mice became significantly hypoglycaemic following the induction of endotoxaemia. While both vehicle- and antagonist-treated animals became significantly hypernatraemic and hyperchloraemic over the course of endotoxaemia, consistent with dehydration and loss of bicarbonate respectively, no change in plasma Na ^+ ^or Cl ^-^ levels was observed in TRPV4 KO mice. Correspondingly, bicarbonate levels decreased in both vehicle- and antagonist-treated mice, but not in TRPV4 KO mice. Both haemoglobin and haematocrit decreased slightly with endotoxaemia, though to an equivalent extent across groups.

Interestingly, naïve TRPV4 KO mice also exhibited trends towards pathological blood biochemistry. Blood pH was significantly decreased in these mice, and they also showed a trend to elevated blood urea concentration, relative to vehicle-treated WT controls. Basal haemoglobin and haematocrit levels were also decreased in naïve TRPV4 KO mice, relative to WT controls, indicative of basal anaemia ( Table 2).

**Table 2.** Blood biochemistry in healthy and endotoxaemic TRPV4 WT and KO mice. Blood gas and biochemistry were measured from venous blood samples by iSTAT point-of-care analyser in either TRPV4 KO mice, or WT mice treated i.p. with vehicle (10% DMSO) or HC-067047 (HC) for 24 h, either under naïve or endotoxaemic (LPS 12.5 mg/kg, i.v., 24 h) conditions. Data are presented as mean ± SEM. *p<0.05, **p<0.01, ***p<0.001, relative to naïve controls; ^#^p<0.05, relative to vehicle-treated controls, 1-way ANOVA + Bonferroni post-hoc test (n = 6–9).

	Vehicle Naïve	HC Naïve	KO Naïve	Vehicle LPS	HC LPS	KO LPS
**Urea** (mmol/L)	5.100 ± 0.51	4.938 ± 0.44	5.417 ± 0.39	22.45 ± 5.55*	18.53 ± 3.81	30.30 ± 4.85***
**pH**	7.314 ± 0.01	7.291 ± 0.01	7.189 ± 0.03 ^#^	7.095 ± 0.03***	7.156 ± 0.02**	7.090 ± 0.04
**Base excess** (mmol/L)	-12.38 ± 0.80	-11.38 ± 1.03	-14.83 ± 0.79	-17.75 ± 1.05	-16.11 ± 1.23	-16.78 ± 2.05
**HCO _3_^-^** (mmol/L)	13.73 ± 0.88	15.41 ± 1.09	13.55 ± 0.94	11.90 ± 0.73	12.88 ± 1.26	13.14 ± 1.74
**PCO _2_** (mmHg)	27.25 ± 2.18	32.06 ± 2.40	36.65 ± 5.20	39.08 ± 3.15	36.90 ± 4.02	42.11 ± 4.99
**TCO _2_** (mmol/L)	14.63 ± 0.92	16.50 ± 1.15	14.67 ± 1.02	13.13 ± 0.74	14.00 ± 1.36	14.33 ± 1.85
**Glucose** (mmol/L)	11.70 ± 1.03	12.36 ± 0.90	10.77 ± 1.10	1.08 ± 0.04***	1.34 ± 0.12***	1.76 ± 0.50***
**Na ^+^** (mmol/L)	150.3 ± 1.7	147.9 ± 1.0	150.2 ± 0.9	155.9 ± 0.9*	154.0 ± 1.1**	150.6 ± 1.0 ^#^
**K ^+^** (mmol/L)	4.088 ± 0.15	4.250 ± 0.19	4.150 ± 0.24	3.950 ± 0.30	3.589 ± 0.32	3.625 ± 0.23
**Cl ^-^** (mmol/L)	121.8 ± 1.3	119.5 ± 0.8	124.0 ± 0.6	130.0 ± 1.6*	128.6 ± 1.0***	124.3 ± 1.6 ^#^
**Anion gap** (mmol/L)	18.50 ± 0.53	17.25 ± 0.94	16.67 ± 0.92	18.50 ± 0.87	16.11 ± 1.11	16.25 ± 1.03
**Haemoglobin** (g/dl)	10.41 ± 0.54	10.93 ± 0.59	8.60 ± 0.25	9.43 ± 0.71	8.96 ± 7.33	9.53 ± 0.59
**Haematocrit** (%PCV)	30.63 ± 1.60	32.13 ± 1.74	25.25 ± 0.75	29.57 ± 1.17	27.88 ± 1.36	28.00 ± 1.72

**Abbreviations:** PCO _2_, partial pressure of CO _2_; TCO _2_, total carbon dioxide; PCV, packed cell volume; HC, HC-067047; KO, TRPV4 knockout; LPS, lipopolysaccharide

Raw Data for Table 2 Blood biochemistry in healthy and endotoxaemic TRPV4 WT and KO mice. Blood gas and biochemistry were measured from venous blood samples by iSTAT point-of-care analyser in either TRPV4 KO mice, or WT mice treated i.p. with vehicle (10% DMSO) or HC-067047 (HC) for 24 h, either under naïve or endotoxaemic (LPS 12.5 mg/kg, i.v., 24 h) conditions. **Abbreviations:** PCO _2,_ partial pressure of CO _2_; TCO _2_, total carbon dioxide; PCV, packed cell volume; HC, HC-067047; KO, TRPV4 knockout; LPS, lipopolysaccharide ( Sand *et al.*, 2015e).

The effect of pharmacological TRPV4 antagonism was also assessed at an earlier time-point in pathogenesis. In contrast with the 24 h time-point, no significant difference in severity score, percentage weight loss, or core temperature was observed between vehicle- and antagonist-treated mice at 6 h post-LPS (data not shown). Blood biochemistry was similarly equivalent between treatment groups at the 6 h time-point ( Table 3).

**Table 3.** Blood biochemistry in healthy and endotoxaemic WT mice treated with TRPV4 antagonist or vehicle. Blood gas and biochemistry were measured from venous blood samples by iSTAT point-of-care analyser in WT mice treated i.p. with vehicle (10% DMSO) or HC-067047 (HC), either under naïve (24-h treatment) or endotoxaemic (LPS 12.5 mg/kg, i.v., 6 h or 24 h) conditions. Data are presented as mean ± SEM. *p<0.05, **p<0.01, ***p<0.001, relative to naïve controls, 1-way ANOVA + Bonferroni post-hoc test (n = 8–9).

	Vehicle Naïve	HC Naïve	Vehicle LPS 6 h	HC LPS 6 h	Vehicle LPS 24 h	HC LPS 24 h
**Urea** (mmol/L)	5.100 ± 0.51	4.938 ± 0.44	7.888 ± 0.51	7.838 ± 0.76	22.45 ± 5.55*	18.53 ± 3.81
**pH**	7.314 ± 0.01	7.291 ± 0.01	7.073 ± 0.03***	7.090 ± 0.02***	7.095 ± 0.03***	7.156 ± 0.02**
**Base excess** (mmol/L)	-12.38 ± 0.80	-11.38 ± 1.03	-19.00 ± 1.18*	-18.63 ± 1.16**	-17.75 ± 1.05	-16.11 ± 1.23
**HCO _3_^-^** (mmol/L)	13.73 ± 0.88	15.41 ± 1.09	11.09 ± 0.89	11.30 ± 1.13	11.90 ± 0.73	12.88 ± 1.26
**PCO _2_** (mmHg)	27.25 ± 2.18	32.06 ± 2.40	38.25 ± 3.28	37.46 ± 4.41	39.08 ± 3.15	36.90 ± 4.02
**TCO _2_** (mmol/L)	14.63 ± 0.92	16.50 ± 1.15	12.13 ± 0.97	12.38 ± 1.22	13.13 ± 0.74	14.00 ± 1.36
**Glucose** (mmol/L)	11.70 ± 1.03	12.36 ± 0.90	2.063 ± 0.52***	1.838 ± 0.42***	1.08 ± 0.04***	1.34 ± 0.12***
**Na ^+^** (mmol/L)	150.3 ± 1.7	147.9 ± 1.0	156.8 ± 0.4**	156.6 ± 0.5***	155.9 ± 0.9*	154.0 ± 1.1**
**K ^+^** (mmol/L)	4.088 ± 0.15	4.250 ± 0.19	3.788 ± 0.20	3.788 ± 0.33	3.950 ± 0.30	3.589 ± 0.32
**Cl ^-^** (mmol/L)	121.8 ± 1.3	119.5 ± 0.8	131.3 ± 1.0***	131.4 ± 0.9***	130.0 ± 1.6*	128.6 ± 1.0***
**Anion gap** (mmol/L)	18.50 ± 0.53	17.25 ± 0.94	18.13 ± 1.11	17.50 ± 0.50	18.50 ± 0.87	16.11 ± 1.11
**Haemoglobin** (g/dl)	10.41 ± 0.54	10.93 ± 0.59	9.44 ± 0.64	9.23 ± 0.85	9.43 ± 0.71	8.96 ± 7.33
**Haematocrit** (%PCV)	30.63 ± 1.60	32.13 ± 1.74	27.75 ± 1.92	29.14 ± 1.71	29.57 ± 1.17	27.88 ± 1.36

**Abbreviations:** PCO _2_, partial pressure of CO _2_; TCO _2_, total carbon dioxide; PCV, packed cell volume; HC, HC-067047; KO, TRPV4 knockout; LPS, lipopolysaccharide

Raw data for Table 3 Blood biochemistry in healthy and endotoxaemic WT mice treated with TRPV4 antagonist or vehicle. Blood gas and biochemistry were measured from venous blood samples by iSTAT point-of-care analyser in WT mice treated i.p. with vehicle (10% DMSO) or HC-067047 (HC), either under naïve (24-h treatment) or endotoxaemic (LPS 12.5 mg/kg, i.v., 6 h or 24 h) conditions ( Sand *et al.*, 2015f).

**Figure 6.**
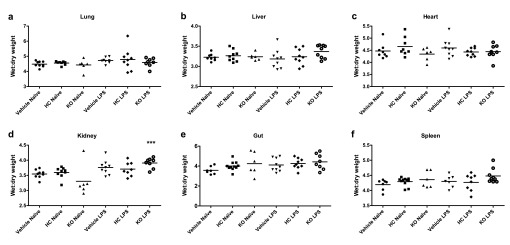
Oedema formation in TRPV4 WT and KO mice. Oedema formation was measured by comparing wet and dry weights of ( **a**) lungs, ( **b**) hearts, ( **c**) upper liver lobes, ( **d**) right kidneys, ( **e**) 1-cm sections of small intestine and ( **f**) whole spleens. WT mice were treated i.p. with vehicle (10% DMSO) or HC-067047 (HC) for 24 h, either under naïve or endotoxaemic (LPS 12.5 mg/kg, i.v., 24 h) conditions. Values for each animal are presented as individual symbols, with horizontal line denoting group mean. ***p<0.001, relative to naïve controls, 1-way ANOVA + Bonferroni post-hoc test (n = 6–9).

Oedema formation was measured by comparing wet and dry weights of various tissues. Surprisingly, treatment with LPS for 24 h did not cause any appreciable oedema formation in WT mice. Endotoxaemic TRPV4 KO mice exhibited marginally elevated oedema formation in the liver and spleen, and significantly increased oedema in the kidney. No difference was observed between vehicle and antagonist-treated mice ( Figure 6).

Raw data for Figure 6 Oedema formation in TRPV4 WT and KO mice was measured by comparing wet and dry weights of various organs. WT mice treated i.p. with vehicle (10% DMSO) or HC-067047 (HC) for 24 h, either under naïve or endotoxaemic (LPS 12.5 mg/kg, i.v., 24 h) conditions ( Sand *et al.*, 2015g).

**Figure 7.**
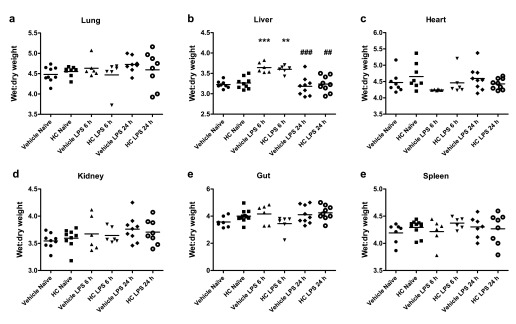
Oedema formation in WT mice treated with TRPV4 antagonist or vehicle. Oedema formation in WT mice treated with TRPV4 antagonist or vehicle. Oedema formation was measured by comparing wet and dry weights of ( **a**) lungs, ( **b**) upper liver lobes, ( **c**) hearts, ( **d**) right kidneys, ( **e**) 1-cm sections of small intestine and ( **f**) whole spleens. WT mice were treated i.p. with vehicle (10% DMSO) or HC-067047 (HC) at the time of LPS (12.5 mg/kg, i.v., 6 or 24 h). Values for each animal are presented as individual symbols, with horizontal line denoting group mean. **p<0.01, ***p<0.001, relative to naïve controls, 1-way ANOVA + Bonferroni post-hoc test (n = 6–9).

The effect of TRPV4 antagonism on oedema formation was also assessed at an earlier time-point in pathogenesis. In contrast with the 24-h time-point, significant liver oedema was observed in both vehicle- and HC-067047-treated mice after 6 h, though this had returned to baseline levels by 24 h post-LPS ( Figure 7). No significant difference in oedema formation was observed in any other tissues, however. No changes in the ratio of heart weight to body weight were observed following the induction of endotoxaemia in any of the animal groups.

Raw Data for Figure 7 Oedema formation in WT mice treated with TRPV4 antagonist or vehicle. Oedema formation was measured by comparing wet and dry weights of organs. WT mice were treated i.p. with vehicle (10% DMSO) or HC-067047 (HC) at the time of LPS (12.5 mg/kg, i.v., 6 or 24 h) ( Sand *et al.*, 2015h).

**Figure 8.**
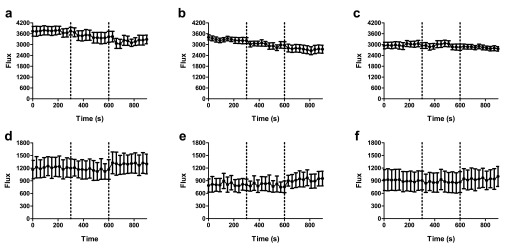
Vasoactive responses to GSK1016790A and vehicle in healthy and endotoxaemic WT mice. Baseline mesenteric blood flow was recorded for 5 min in naïve ( **a**– **c**) and endotoxaemic ( **d**– **f**) mice, 24 h after injections of LPS. Vehicle (V; 2% DMSO in saline) was then administered as an aerosolised spray, followed by administration of GSK1016790A (G; 1 µM) 5 min later. Blood flow was recorded in ( **a** & **d**) 1 ^st^ order, ( **b** & **e**) 2 ^nd^ order and ( **c** & **f**) 3 ^rd^ order mesenteric vessels. Data are presented as mean ± SEM. *p<0.05, area under the curve relative to baseline, 1-way ANOVA + Bonferroni post-hoc test (n = 5).

In order to assess changes in TRPV4 activity *in vivo* after the induction of endotoxaemia, 1 µM GSK1016790A and vehicle (2% DMSO in pre-warmed saline) were sequentially applied to the exposed mesenteric bed by aerosolised spray. No significant responses to GSK1016790A were observed in the mesenteric beds of naive mice: both vehicle and GSK1016790A caused a non-significant decrease in blood flow, likely to reflect evaporative cooling ( Figure 8a–c and Table 4). In LPS-treated mice, on the other hand, GSK1016790A caused an increase in blood flow ( Figure 8d–f) that was significant in 2 ^nd^ and 3 ^rd^ order mesenteric vessels ( Table 4).

**Table 4.** Change in mesenteric blood flow following topical TRPV4 activation in naïve and septic mice. Baselines (BL), and responses to aerosolised vehicle (Veh; 10% DMSO in pre-warmed saline) and GSK1016790A (GSK; 1 µM) were recorded over sequential 5-min periods. Data are presented as mean percentage change in area under the curve over 5-min recording period. *p<0.05, **p<0.01, relative to vehicle response in the same mouse, paired 2-tailed Student’s T-test; ^#^p<0.05 relative to corresponding response in naïve controls, 1-way ANOVA + Bonferroni post-hoc test (n = 5).

	Naive			LPS 24 h		
	*1 ^st^ order*	*2 ^nd^ order*	*3 ^rd^ order*	*1 ^st^ order*	*2 ^nd^ order*	*3 ^rd^ order*
BL−Veh	-7.33 ± 3.10%	-8.39 ± 1.73%	-1.10 ± 0.32%	-3.00 ± 2.77%	-1.18 ± 1.51%	-2.22 ± 3.87%
Veh−GSK	-7.61 ± 1.30%	-8.46 ± 3.27%	-3.42 ± 2.05%	+12.11 ± 6.19%	+14.71 ± 6.70%* ^,#^	+11.73 ± 8.72%**

## Discussion

Based on observations that excessive TRPV4 activation can cause profound hypotension, endothelial failure and circulatory collapse ( Willette *et al.*, 2008), we hypothesised that enhanced TRPV4 activity may contribute to haemodynamic and vascular dysfunction during sepsis. Numerous endogenous agonists and regulators of TRPV4, including anandamide ( Varga *et al.*, 1998), arachidonic acid and its metabolites ( Bruegel *et al.*, 2012), protein kinase A ( Yang *et al.*, 1997), shear stress and temperature have been shown to be upregulated or altered in sepsis. It is conceivable therefore, that inflammation-induced upregulation of endogenous factors could contribute to sepsis-associated circulatory failure via excessive TRPV4 activation. By extension, blockade of TRPV4 activity may be expected to attenuate the circulatory dysfunction that underlies sepsis pathogenesis. To test this hypothesis we used both TRPV4 KO mice and the selective TRPV4 antagonist, HC-067047.

Consistent with previous reports ( Nishijima *et al.*, 2014; Zhang *et al.*, 2009), TRPV4 KO mice exhibited lower basal arterial pressure than WT counterparts during both daytime and night-time periods. This is surprising given that TRPV4 has been implicated in both nitric oxide- (NO) and endothelium-derived hyperpolarising factor (EDHF)-mediated vasodilatory responses to physical and chemical stimuli in both conduit and resistance arteries ( Adapala *et al.*, 2011; Hartmannsgruber *et al.*, 2007; Köhler *et al.*, 2006; Loot *et al.*, 2008; Ma *et al.*, 2013; Mendoza *et al.*, 2010; Rath *et al.*, 2012 Sonkusare *et al.*, 2012; Zhang *et al.*, 2009). A possible explanation is that loss of TRPV4 leads to compensatory upregulation of alternative vasodilatory pathways during development. Although studies of isolated vessels from TRPV4 KO mice have shown impaired vasodilatation in response to a range of physiological stimuli ( Earley *et al.*, 2009; Zhang *et al.*, 2009), the effects of isolation and *ex vivo* culture may have altered the mechanisms regulating vascular tone. Alternatively, it is possible that loss of TRPV4 expression in vascular smooth muscle may represent loss of a vasoconstrictor mechanism. While numerous studies have suggested that TRPV4 in vascular smooth muscle couples with hyperpolarising K ^+^ channels to induce vasodilatation ( Earley *et al.*, 2005; Earley *et al.*, 2009), activation of TRPV4 simultaneously across multiple cells (as opposed to very localised activation) may trigger Ca ^2+^-dependent vasoconstriction to maintain a basal level of vascular tone. Indeed, TRPV4-mediated vasoconstriction has previously been demonstrated in the lung, where TRPV4 gene ablation suppresses the development of chronic hypoxic pulmonary hypertension ( Xia *et al.*, 2013).

Given that TRPV4 exhibits tonic activity both in heterologous expression systems ( Strotmann *et al.*, 2000) and in primary endothelial cells ( Sonkusare *et al.*, 2012), its blockade may be expected to cause acute hypertension. While systemic administration of HC-067047 did induce a transient increase in blood pressure under basal conditions, a similar effect was observed in vehicle-treated animals, suggesting that the transient hypertension may have been a stress response to manual handling. In accordance with these data, previous studies have shown that administration of a different TRPV4 antagonist – GSK 2193874 – does not significantly alter blood pressure after either acute intravenous administration or repeated oral gavage ( Thorneloe *et al.*, 2012).

The lack of any dramatic effect on blood pressure is consistent with a previous *ex vivo* study showing that blockade of the TRPV4-cytochrome P450 epoxygenase axis only inhibits flow-induced vasodilatation in the presence of nitric oxide synthase (NOS) and prostacyclin (PGI _2_) antagonists ( Loot *et al.*, 2008). In other words, blockade of one endothelium-dependent vasodilatory pathway can lead to an acute increase in activity of a compensatory pathway. Elevated activity in an alternative (and perhaps more potent) vasodilatory pathway following loss of TRPV4 activity may also explain the subsequent night-time decreases in blood pressure following HC-067047 administration, which were not evident in vehicle-treated mice (as well as the trend towards hypotension in TRPV4 KO mice). A compensatory increase in, for example, NOS activity and PGI _2_ production could also account for the night-time decreases in heart rate following TRPV4 antagonism, since both are known to produce negative chronotropy ( Chiba & Malik, 1980; Feron *et al.*, 1998). Further experiments are necessary to determine whether the activity of other vasorelaxant pathways is altered in TRPV4 knockout animals.

Contrary to expectations, neither TRPV4 gene deletion nor pharmacological antagonism attenuated LPS-induced haemodynamic dysfunction. In fact, both HC-067047-treated and TRPV4 KO mice exhibited a trend towards exaggerated hypotension that was most evident in measurements of systolic pressure and pulse pressure. The exaggerated systolic hypotension and greater decrease in pulse pressure observed in both antagonist-treated and KO mice may be indicative of reduced cardiac output and left ventricular dysfunction, though these parameters were not measured in this study. Reduced water intake in these animals prior to the induction of sepsis may have contributed to hypovolaemia, decreasing cardiac output. Alternatively, it is possible that elevated vascular NO production (in response to loss of other TRPV4-dependent vasodilator mechanisms) could have increased aortic compliance in these animals, leading to greater systolic hypotension and a narrower pulse pressure.

Overall, the haemodynamic consequences of endotoxaemia appeared to be worse in mice lacking TRPV4: hypotension was slightly exaggerated, and the baroreceptor reflex (compensatory tachycardia in response to a fall in blood pressure) was almost entirely absent in TRPV4 KO mice. Given that NO is thought to antagonise the positive chronotropic effects of increased sympathetic drive ( Feron *et al.*, 1998), it is possible that excessive NO production in TRPV4 KO mice could account for the bradycardia following LPS administration.

Low mesenteric flow is known to correlate strongly with multiple organ failure and mortality, both in animal models ( Baykal *et al.*, 2000) and in human patients ( Takala, 1996). Clinically, gut ischaemia is known as the ‘motor of multiple organ failure’ ( Carrico *et al.*, 1986), probably because impaired mesenteric blood flow is associated with intestinal hyperpermeability. This breakdown in barrier function facilitates the leakage of endotoxins and microorganisms into the lymphatic and cardiovascular circulation, which can exacerbate the inflammatory response ( Sautner *et al.*, 1998). Furthermore, gastric perfusion is known to correlate well with sublingual perfusion ( Marik, 2001) – a robust indicator of outcome in patients ( De Backer *et al.*, 2010). Despite numerous reports of an important role for TRPV4 in mediating agonist- and flow-induced vasoactive responses in small resistance vessels ( Adapala *et al.*, 2011; Earley *et al.*, 2009; Köhler *et al.*, 2006; Ma *et al.*, 2013; Mendoza *et al.*, 2010; Sonkusare *et al.*, 2012; Zhang *et al.*, 2009), we did not observe any marked changes in mesenteric blood flow in antagonist-treated or TRPV4 KO mice. There was, however, a trend towards improved flow in 1 ^st^ and 2 ^nd^ order vessels in endotoxaemic HC-067047-treated mice. Of note, perhaps, is the observation that antagonist-treated mice exhibited a trend towards exaggerated hypotension following LPS administration, but marginally better mesenteric perfusion; these results are consistent with the notion that stabilisation of arterial pressure during sepsis may occur at the expense of microcirculatory flow ( Sand *et al.*, 2015).

The exact half-life of HC-067047 has not been reported, though plasma levels are known to exceed the IC _50_ for some hours after i.p. administration ( Everaerts *et al.*, 2010), so we also investigated the effect of TRPV4 antagonism on mesenteric blood flow at an earlier time-point. At 6 h post-LPS, there was a trend towards lower blood flow in antagonist-treated mice, consistent with a vasodilatory role for TRPV4 in the mesenteric vasculature, though this was not statistically significant.

While TRPV4 is known to dilate small resistance vessels (such that its blockade may be expected to decrease mesenteric blood flow) its activation has also been associated with increased endothelial permeability and oedema formation, which would impair perfusion ( Willette *et al.*, 2008). These divergent effects on microvascular blood flow may account for the reverse in trends between 6 h and 24 h treatment with LPS. Nonetheless, our data do not support a prominent role for TRPV4 in regulating mesenteric blood flow in this model, either under basal or endotoxaemic conditions. It is likely that activity in compensatory pathways is increased both acutely and chronically to compensate for its loss.

In contrast to numerous studies demonstrating TRPV4-mediated dilatation of mesenteric arteries ( Adapala *et al.*, 2011; Earley *et al.*, 2009; Köhler *et al.*, 2006; Ma *et al.*, 2013; Mendoza *et al.*, 2010; Sonkusare *et al.*, 2012; Zhang *et al.*, 2009) topical application of GSK1016790A did not induce any marked changes in blood flow in naïve mice. Despite the relatively high concentration used (1 µM), it is possible that owing to the aerosolised delivery method (which may be prone to evaporation) insufficient concentrations of the agonist contacted vascular receptors. While TRPV4 is expressed in vascular smooth muscle ( Earley *et al.*, 2005), the compound may not have reached the luminal side of the vessel to induce endothelium-dependent vasodilatation. Additional experiments using higher concentrations or a different delivery method are required to elucidate this further. The observation that GSK1016790A caused an increase in blood flow in LPS-treated mice may indicate inflammatory TRPV4 sensitisation, with small amounts of luminal GSK1016790A now able to induce vasodilatation, in line with our overall hypothesis.

Interestingly, naive TRPV4 KO mice exhibited signs of pathology. Blood pH was significantly lower in these animals compared with WT counterparts; base excess was correspondingly reduced, and partial pressure of CO _2_ elevated, indicative of respiratory acidosis. These data are consistent with a potential acid-sensing role for TRPV4 reported previously ( Suzuki *et al.*, 2003). The observation that blood pH was also slightly reduced in naive HC-067047-treated mice relative to vehicle-treated controls, suggests that TRPV4 may be involved in maintaining acid-base homeostasis, though the mechanism by which this occurs is unclear.

Following the induction of endotoxaemia, TRPV4 KO mice exhibited a more dramatic increase in plasma urea concentration than WT mice, consistent with a role for TRPV4 in regulating renal function ( Liedtke & Friedman, 2003). Although not measured in this study, it is possible that loss of TRPV4 activity may result in impaired renal circulation. TRPV4 KO mice also exhibited the lowest blood pH of all groups during sepsis, but this was not significantly different from the mildly acidotic basal pH in these animals. Both antagonist- and vehicle-treated WT mice, in contrast, exhibited a significant decline in pH between basal and septic conditions, though the magnitude of the decrease was less pronounced in HC-067047-treated animals. This suggests that blockade of TRPV4 activity may attenuate the development of metabolic acidosis. Consistent with this notion, base excess was slightly lower and bicarbonate levels slightly higher in both antagonist-treated and TRPV4 KO mice, relative to vehicle-treated controls.

Both vehicle- and antagonist-treated WT animals became severely hyperchloraemic over the course of sepsis. Fluid loss, and perhaps the administration of saline resuscitation at the time of sepsis, could have contributed to this finding. Hyperchloraemic metabolic acidosis (in which serum Cl ^-^ levels are elevated, bicarbonate reduced and blood pH is low) is also indicative of kidney dysfunction ( Walker, 1990). Surprisingly, given the elevated plasma urea levels, TRPV4 KO mice did not exhibit LPS-induced hyperchloraemia or hypernatraemia.

Following reports linking excessive TRPV4 activation to endothelial failure and oedema formation ( Alvarez *et al.*, 2006; Willette *et al.*, 2008) we aimed to establish the role of TRPV4 in oedema formation during endotoxaemia – a condition characterised by increased endothelial permeability ( Bridges & Dukes, 2005). No marked changes in the ratio of tissue wet-to-dry weight were observed in WT mice following 24 h treatment with LPS; only the lung and the kidney exhibited trends towards increased plasma extravasation, and values were not different between vehicle- and antagonist-treated mice. Although these data indicate that oedema is not present at this time-point in endotoxaemia pathogenesis, it is possible that the method used is insufficiently sensitive to detect small changes in plasma extravasation and interstitial fluid volume. We did observe evidence of oedema formation in the kidneys of LPS-treated TRPV4 KO mice (there were also trends towards increased wet-to-dry weight ratios in the livers and spleens of these animals). This was surprising given that these mice did not exhibit an altered ionic balance, and furthermore, because activation of TRPV4 has been associated with increased vascular permeability ( Alvarez *et al.*, 2006; Willette *et al.*, 2008). Nonetheless, it appears that deletion of the TRPV4 gene is associated with slightly exaggerated oedema formation in the kidney, liver and spleen following LPS administration, though it is not clear what mechanism may be involved.

The dose of HC-067047 used in this study (10 mg/kg) was chosen based on the only two previous reports of its administration *in vivo* ( Everaerts *et al.*, 2010; Materazzi *et al.*, 2012). While the effects of systemic TRPV4 antagonism were not dramatic, use of higher concentrations was not possible: intraperitoneal injection of 100 mg/kg HC-067047 has been shown to cause obvious adverse effects, including hunching and piloerection ( Everaerts *et al.*, 2010), and higher concentrations of DMSO could potentially be toxic. Repeated dosing may represent a more viable option for blood flow recording or biochemical analysis, though repeated manual handling and intraperitoneal injections would certainly preclude meaningful haemodynamic recording. Supplementation of drinking water with orally available TRPV4 antagonists ( Thorneloe *et al.*, 2012) would be required to further elucidate the effects of TRPV4 blockade on cardiovascular function in endotoxaemia or sepsis.

Nonetheless, plasma levels of the compound have been shown to remain above the IC _50_ for more than 2 h after intraperitoneal administration of 10 mg/kg HC-067047 ( Everaerts *et al.*, 2010), suggesting that any prominent role for TRPV4 in sepsis-associated cardiovascular dysfunction would at least have been evident in the early phase of sepsis. The fact that no major changes in haemodynamics, mesenteric blood flow, blood biochemistry or tissue oedema were observed at 6 h post-LPS, suggests either that TRPV4 does not play a major role in cardiovascular regulation during endotoxaemia, or that other pathways can be upregulated acutely to compensate for its loss. However, further studies in which research conditions are optimised may yet uncover evidence of a vasoregulatory role for TRPV4 in sepsis.

Overall, our data do not support a major role for TRPV4 in sepsis-associated cardiovascular dysfunction. It is possible that upregulation of alternative vasodilatory pathways may compensate for its loss both acutely and chronically, and that changes would only be evident on blockade of, for example, NO- and PGI _2_-dependent pathways. Alternatively, since plasma osmolality is known to increase in sepsis ( Jochberger *et al.*, 2009), and hypertonicity inhibits TRPV4 activity ( Strotmann *et al.*, 2000), it is possible that under septic conditions, TRPV4 activity is inhibited such that gene deletion or pharmacological antagonism will produce no further effect. Regardless of underlying mechanisms, based on data obtained in this study, TRPV4 does not appear to be a viable target in the treatment of sepsis-associated cardiovascular dysfunction.

## Data availability

*F1000Research*: Dataset 1. Raw Data for Figure 2, 10.5256/F1000Research.6298.d45231 ( Sand *et al.*, 2015a).

*F1000Research*: Dataset 2. Raw Data for Figure 3, 10.5256/F1000Research.6298.d45232 ( Sand *et al.*, 2015b).

*F1000Research*: Dataset 3. Raw Data for Figure 4, 10.5256/F1000Research.6298.d45233 ( Sand *et al.*, 2015c).

*F1000Research*: Dataset 4. Raw Data for Figure 5, 10.5256/F1000Research.6298.d45234 ( Sand *et al.*, 2015d).

*F1000Research*: Dataset 5. Raw Data for Table 2, 10.5256/F1000Research.6298.d45237 ( Sand *et al.*, 2015e).

*F1000Research*: Dataset 6. Raw data for Table 3, 10.5256/F1000Research.6298.d45238 ( Sand *et al.*, 2015f).

*F1000Research*: Dataset 7. Raw data for Figure 6, 10.5256/F1000Research.6298.d45235 ( Sand *et al.*, 2015g).

*F1000Research*: Dataset 8. Raw Data for Figure 7, 10.5256/F1000Research.6298.d45236 ( Sand *et al.*, 2015h).
