# Supplementary material for: Blockade or deletion of transient receptor potential vanilloid 4 (TRPV4) is not protective in a murine model of sepsis
Source: F1000Res. 2015 Apr 20;4:93. [Version 1] doi: 10.12688/f1000research.6298.1 (PMC4448752; doi:10.12688/f1000research.6298.1)
Supplement: Peer review history

# Peer review: Referee response for version 1

**DOI:** 10.5256/f1000research.6755.r8382

**Published:** 2015-05-14

**Authors:** David X. Zhang

**Affiliations:** 1 Department of Medicine, USA Cardiovascular Center, Medical College of Wisconsin, Milwaukee, WI, USA

Sand *et al. *examined the hypothesis that loss of TRPV4 function prevents cardiovascular dysfunction in sepsis. Using TRPV4-knockout mice and animals treated with TRPV4 inhibitor (HC-067047), the authors did not find a protective role of TRPV4 deletion/blockade in the model of LPS-induced endotoxaemia. Although the results are not supportive of the original hypothesis, the study is well-designed and provides further insight into the role of TRPV4 *in vivo *in the regulation of cardiovascular function.

Consistent with previous reports, TRPV4 KO animals (a different colony generated by Liedtke & Friedman) exhibited lower basal arterial pressure than WT controls during both daytime and night-time periods while acute administration of TRPV4 antagonist HC-067047 did not significantly alter blood pressure as compared to vehicle. The minor impact of TRPV4 activity on the regulation of blood pressure is in contrast to the important role of this channel in vasodilatory response to physical and chemical stimuli in conduit and resistance arteries. The authors proposed that this discrepancy could be due to the activation of compensatory pathways in the absence of TRPV4 function. It also remains to be explored whether TRPV4 function in the vascular tissue could be counteracted by its activity in other tissues such as those involving in baroreceptor reflex or in long-term sodium/water balance by using tissue-specific KO.

The endotoxaemic mice used in this study showed only small decrease in blood pressure over baseline, e.g. an average reduction in MAP of 7.6 mmHg during daytime and 12.9 mmHg at night-time in WT. The animals also exhibited reduced mesenteric blood flow and lower core body temperature. Together, these signs seem to indicate a hypodynamic state of sepsis, and the obtained results might underestimate or overlook hemodynamic effects of TRPV4 activation occurred during an early hyperdynamic stage of sepsis. This potential limitation could be discussed in the manuscript.

It was also noted that endotoxaemic animals have very low ambulatory activity throughout 24-h period after LPS and a marked reduction in blood glucose (by as much as 10-fold over baseline). Are these alterations also found in other similar septic murine models? A reduced ambulatory activity at night-time could contribute to observed greater reduction in blood pressure during this period after LPS challenge.

Other points

1. Table 1, column 1. It seems that the comparison should be “BL2-BL1”, “LPS-BL1”, and “LPS-BL2”.
2. Figures 4 and 8, mesenteric blood flow. A large variation in AUC values is noted between the two figures.

I have read this submission. I believe that I have an appropriate level of expertise to confirm that it is of an acceptable scientific standard.

# Peer review: Referee response for version 1

**DOI:** 10.5256/f1000research.6755.r8381

**Published:** 2015-05-13

**Authors:** Scott Earley

**Affiliations:** 1 Department of Pharmacology, University of Nevada School of Medicine, Reno, NV, USA

Sand *et al. *examinesd the hypothesis that activity of the Ca2 ^+^-permeable the transient receptor potential (TRP) channel TRPV4 contributes to vascular dysfunction in an animal model of sepsis. The study utilized selective pharmacology and TRPV4 knockout animals to determine if loss of TRPV4 expression would be protective against sepsis induced by injection of lipopolysaccharide (LPS). Contrary to expectations, loss or blockade of TRPV4 had very little effect on LPS-induced hypotension. Similarly, reduction in mesenteric blood flow following LPS administration did not differ between treatment groups. LPS (24 hours) did not induce significant edema formation in control mice, but did cause edema in the kidneys of TRPV4 knockout mice. Edema was present in some tissues at an earlier (6 hours) time point but this response was not affected by TRPV4 block. Administration of the TRPV4 agonist GSK1016790A caused an increase in mesenteric blood flow in 2 ^nd^ and 3 ^rd^ order mesenteric arteries in LPS but not control animals.

This study is clearly written and the work was well designed and properly executed. The guiding hypothesis - block of TRPV4 channels would diminish vascular effects of sepsis - was based on an important study that showed circulatory collapses following systemic administration of a TRPV4 activating drug. This idea is entirely logical and of tremendous interest. Indeed, TRPV4 block appears to improve pulmonary edema associated with heart failure in rodents ( Thorneloe *et al., *2012). However, the current findings do not indicate that block of TRPV4 is beneficial during sepsis. These disparate observations may reflect important differences between the pulmonary and systemic endothelium.

I have read this submission. I believe that I have an appropriate level of expertise to confirm that it is of an acceptable scientific standard.
